# Transgenerational effects of paternal heroin addiction on anxiety and aggression behavior in male offspring

**DOI:** 10.1186/s12993-016-0107-y

**Published:** 2016-08-31

**Authors:** Mohd Zaki Farah Naquiah, Richard Johari James, Suraya Suratman, Lian Shien Lee, Mohd Izhar Mohd Hafidz, Mohd Zaki Salleh, Lay Kek Teh

**Affiliations:** 1 Integrative Pharmacogenomics Institute (iPROMISE), Level 7, FF3, Universiti Teknologi MARA Selangor, Puncak Alam Campus, 42300 Bandar Puncak Alam, Selangor Malaysia; 2 Faculty of Pharmacy, Universiti Teknologi MARA Selangor, Puncak Alam Campus, 42300 Bandar Puncak Alam, Selangor Malaysia; 3 Comparative Medicine and Technology Unit, Institute Bioscience, Universiti Putra Malaysia, 43400 Serdang, Selangor Malaysia

**Keywords:** Transgenerational effects, Paternal heroin addiction, Male offspring, Anxiety, Aggression

## Abstract

**Background:**

Heroin addiction is a growing concern, affecting the socioeconomic development of many countries. Little is known about transgenerational effects on phenotype changes due to heroin addiction. This study aims to investigate changes in level of anxiety and aggression up to four different generations of adult male rats due to paternal exposure to heroin.

**Methods:**

Male Sprague–Dawley rats were exposed with heroin intraperitoneally (i.p.) twice-daily for 14 days with increasing dosage regimen (F0-heroin). Male Sprague–Dawley rats (6-weeks-old) were divided into: (1) heroin exposed group (F0-heroin) and (2) control group treated with saline solution (F0-control). The dosage regime started with the lowest dose of 3 mg/kg per day of heroin followed by 1.5 mg/kg increments per day to a final dose of 13.5 mg/kg per day. Offspring were weaned on postnatal day 21. The adult male offspring from each generation were then mated with female-naïve rats after 2 weeks of heroin absence. Open field test and elevated plus maze test were used to study the anxiety level, whereas resident intruder test was used to evaluate aggression level in the addicted male rats and their offspring.

**Results:**

Heroin exposure in male rats had resulted in smaller sizes of the litters compared to the control. We observed a higher anxiety level in the F1 and F2 progenies sired by the heroin exposed rats (F0) as compared to the control rats. Paternal heroin exposure also caused significantly more aggressive offspring in F1 compared to the control. The same pattern was also observed in the F2.

**Conclusion:**

Our results demonstrated that the progenies of F1 and F2 sustained higher levels of anxiety and aggression which are due to paternal heroin exposure.

## Background

Substance abuse and addiction is one of the serious public health concerns around the world. As reported by The World Drug Report [1], the global prevalence of opiates abusers is 16.5 million worldwide. In Malaysia, heroin is the most abused drugs with a percentage of 64.84 % and males constitute the biggest percentage of abusers each year [2].

The impact of various psychoactive substances on aggressive behavior had been studied previously, due to the documented rise in drug abusers involved in crime. Several drug related factors may contribute to this occurrence, e.g. pharmacological effects of the drug, substance-induced psychological or biological changes, and withdrawal effects [3]. Occasional use of opiates results in pleasure and euphoria [4, 5]. However, chronic exposure to heroin leads to complex changes in mood and behavior, and its abrupt withdrawal may cause adverse after effects i.e. elevated aggression [6] and heightened pain sensitivity [7].

Over the last few decades, there have been several studies of how parental drug abuse may affect their progenies. While most of these studies focused on susceptibility to drug use, others have looked into behavioral, molecular and physiological changes in the offspring. Among them were studies looking at the impact of maternal opioid use on fetal development [8–10]. These studies documented long term consequences on fetal cognitive function including gender-specific modifications in specific emotional and social behaviors [11, 12]. Byrnes et al. [11] has also demonstrated an upregulation of dopamine and opioid-related genes in both mother and child, indicating transgenerational epigenetic effect. A delayed onset of puberty was observed in adolescent male rats exposed to morphine [13]. Mating between the male rats treated with heroin and naïve female rats resulted in smaller litter size. Upon maturation to the adult stage, the offspring displayed significant alteration in endocrine parameters, including gender specific changes in the adrenal weights, luteinizing hormone, and hypothalamic b-endorphin [14].

This study aims to examine the effects of paternal exposure to heroin on anxiety and aggression in the offsprings up to three generations (first generation—F1, second generation—F2 and third generation—F3).

## Methods

### Animals

A total of 64 male and 48 female Sprague–Dawley rats weighing 130–150 g were used. For F0, rats were divided into two experimental groups, with eight rats per group (heroin and control (saline) group). Rats were allowed to acclimatize for 2 weeks prior to experimental procedure and were placed in individual cages. Throughout the experiment, rats were maintained in a temperature and humidity controlled environment (24 °C ± 3.55 ± 1 %) in a 12:12 h light: dark cycle (lights on at 7:00 a.m.) and were given fresh water and standard rat chow ad libitum. All experiments were performed in accordance with the guidelines and approval from the Animal Ethics Committee of Universiti Teknologi MARA (UiTM) (46/2014).

### Drug preparation

Heroin hydrochloride was obtained from LIPOMED (Switzerland). Heroin of 1 mg/ml concentration was prepared daily before injection by dissolving in 0.9 % normal saline heated to 40–50 °C.

### Heroin exposure

Intraperitoneal injections with heroin were given to F0-heroin (F0-H) rats twice daily at 9:00 a.m. and 6:00 p.m., with a 27 gauge 1/2 inch (12 mm) needle for 14 consecutive days. A dose of 3 mg/kg on the first day and then increasing by 1.5 mg/kg per day to a final dose of 13.5 mg/kg per day were administered to the rats [15]. Escalating dose regimen was adopted in order to mimic the different phases of addiction in human. The control rats were injected with the same amount of saline.

### Breeding

**Fig. 1:**
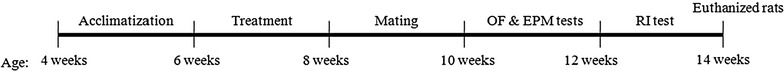
A schematic representation of the experimental regimen for F0. After acclimatization rats were subjected to heroin administration for 2 weeks. The rats were then mated with naïve female rats before the anxiety and aggression test started at week 10

**Fig. 2:**
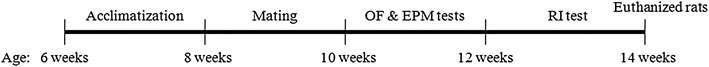
A schematic representation of the experimental regimen for F1 and F2. After acclimatization, rats were mated with naïve female rats at week 8 before being tested with anxiety and aggression at week 10

**Fig. 3:**
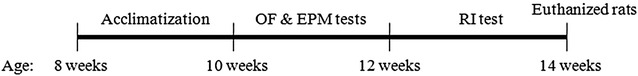
A schematic representation of the experimental regimen for F3. After acclimatization, rats were being tested with anxiety and aggression at week 10

F0 rats were mated with heroin-naïve female rats at 2 weeks post heroin regime (Fig. 1). Withdrawal symptoms were observed during the 2-week period and female rats were introduced as withdrawal symptoms cease. Behavioral tests were then conducted on the rats before they were euthanized on week 11. Male pups were reared by their biological mothers and weaned on postnatal day 21. One male rat was randomly selected from each mother for behavioral testing, and was placed in individual cages each (n = 8). After the F1 rats reached sexual maturation, they were then mated with heroin-naïve female rats to produce F2 (Fig. 2). Behavioral testing commenced at 10 weeks of age. These procedures were then repeated in F2. F2 offspring (F3) were then tested with behavior tests at the age of 10 weeks (Fig. 3). All animals were raised in a similar and controlled environment across all generations.

### Behavior testing

#### Elevated plus maze (EPM) apparatus

The elevated plus maze was constructed of wood and consisted of two open arms, OA (50 × 10 cm) and closed arms, CA (50 × 10 × 40 cm) set in a plus shape. The maze was elevated 50 cm from the floor with an open roof. The activities of the rats were recorded by an overhead camera attached on the ceiling and scoring was analyzed using ANY-maze Video Tracking System software (ANY-maze, Stoelting Co.). The test started with the rats being placed at the center region, facing the OA and were allowed to explore the maze for 5 min. The number of entries to the OA (the percentage of the total number of arm entries) and the total time spent in these arms of the maze were taken as an anxiety index (the higher the index, the lower the anxiety). To measure locomotion, the total number of entries for each arm was taken. An entry was counted when the four paws of the rats were placed in the arms. The maze was cleaned with 70 % ethanol between each trial.

#### Open field (OF) apparatus

To further analyze the anxiety behavior, the animals were tested in the OF for 5 min. The test was carried out in an arena (50 × 50 × 30 cm) divided into 25 squares (10 × 10 cm). Each of the rats was placed in the center and was allowed to explore the arena for 5 min and the time spent in the center of the arena was recorded. After 5 min, the rat was then returned to its home cage and the open field was cleaned with 70 % ethyl alcohol and permitted to dry between tests.

#### Resident-intruder test

In this test, the resident male rats were housed in observation cages (80 × 55 × 50 cm) with a female rat as companion for a week before the test begins to facilitate territorial behavior. As territoriality is based on the presence of olfactory cues, the bedding of the cage was not changed during the week prior to testing. An hour before the test started, the companion female was removed. New male rat (intruder) was then introduced into the resident rat’s cage and the test was started. The resident’s reaction, i.e. non-social activities (attention, rear, sniff, walk, and body care), social activities (approach, follow, walk away, social sniff, genital sniff, and mount), and aggression (tail swish, lateral threat, bite, and clinch/fight), towards the intruder rat was recorded over 10 min. After the test was completed, the intruder rat was removed and the male resident was reunited with its female companion.

#### Statistical analysis

Data are presented as mean ± SEM normal distribution was tested using Kolmogorov–Smirnov test. The data were compared between groups in the same generation using student’s *t* test. One-way ANOVA followed by Dunnett’s or Tukey’s post hoc tests were used for comparison within group for the variables studied in the behavioral test. Statistical significance were set at *p* < 0.05. Statistical parameters were determined using SPSS 20.0 (IBM Corporation, Armonk, NY, USA).

## Results

### Effects of paternal heroin exposure on litter size and body weight of first and second generation pups

The effects of paternal heroin exposure in F0 were apparent in the mean litter size of the first generation (Table 1). Number of offspring in heroin exposed male rat was significantly fewer than that of control group (p = 0.001, 95 % CI 3.07, 8.18). However, this pattern was not observed in second and third generations of the heroin exposed rats.

**Table 1:** Average number of offspring for four generations of the experimental group

Generations	Average number of offspring	
	Control	Heroin
F0	11 ± 0.44	6 ± 1.04*
F1	11 ± 0.48	12 ± 0.32
F2	10 ± 0.29	10 ± 0.39
F3	11 ± 0.27	10 ± 0.33

This table represents the mean pups count per litter at postnatal day 21 (PND21) and postnatal day 90 (PND90)

* Statistically significant difference of p < 0.05 as compared to the control group

### Effects of paternal heroin exposure on behavior of rats in the elevated plus-maze (EPM)

**Fig. 4:**
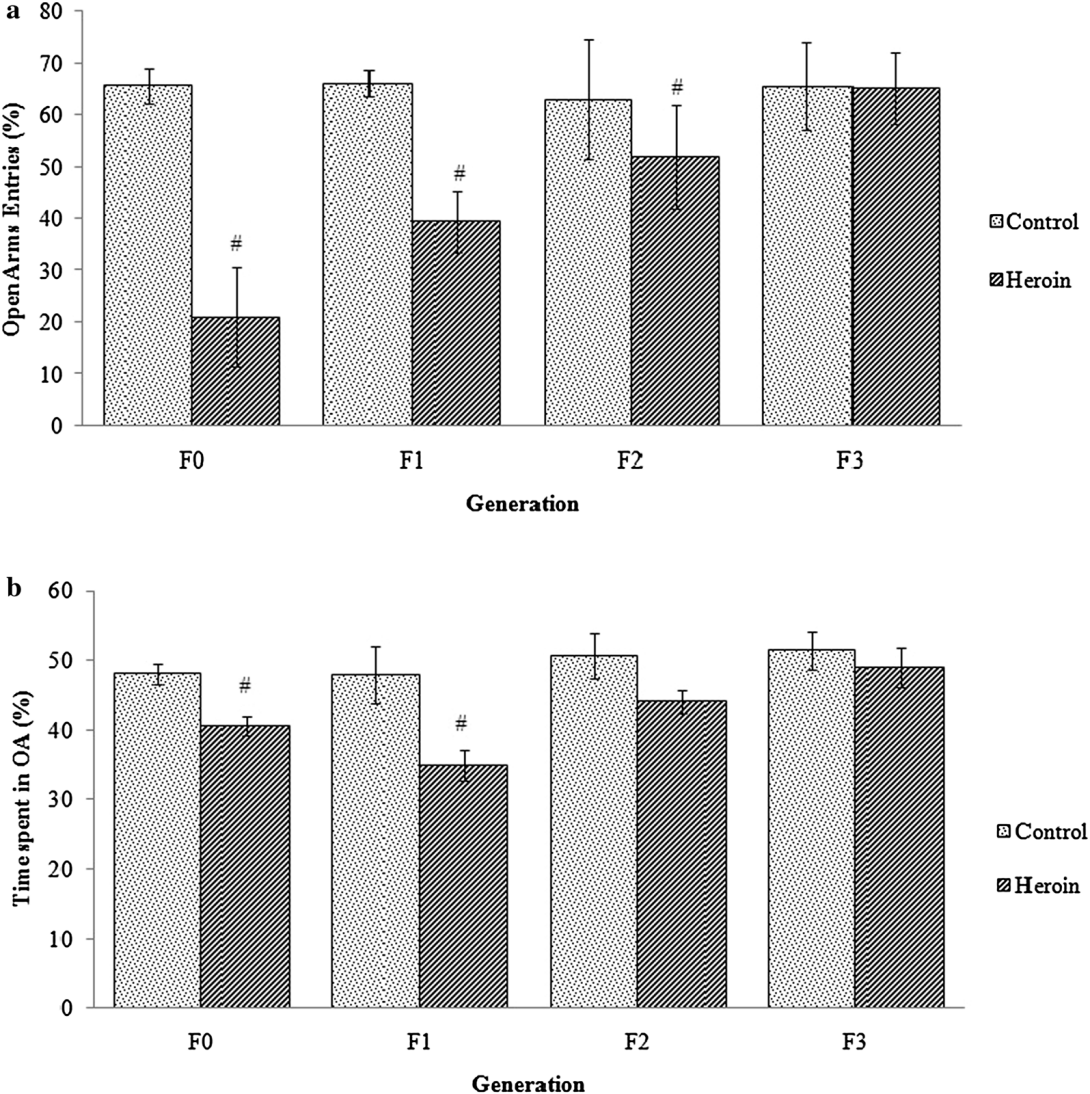
Percentage of entries (**a**) and percentage of time spent in the open arms (**b**) of the elevated plus maze. Values are mean ± SEM. ^#^Statistically significant difference compared to the control group (*p* < 0.05)

As depicted in Fig. 4a, heroin exposure in F0 resulted in a significantly lower percentage of the open arms entries (p = 0.001, 95 % CI 22.99, 66.1), which indicates anxiety effect of heroin withdrawal. In addition, heroin exposed rats spent shorter time in the open arm of the EPM (p = 0.001, 95 % CI 6.90, 15.5). A similar effect was found in the percentage of entries into the open arm of the maze in the first generation offspring of heroin exposed rats (F1-Heroin) compared to the offspring of vehicle-treated group (p = 0.001, 95 % CI 32.87, 60.52). Consistent with these results, rats of the F1-Heroin had a lower percentage of time spent in the open arms of the maze in Fig. 4b when compared to offsprings of control group (F1-control) (p = 0.001, 95 % CI 49.05, 69.41). Interestingly, the second generation (F2) of the male addicted rats exhibit similar pattern with the first generation with a lower OA entries in F2-heroin (p = 0.001, 95 % CI 3.38, 10.41) as compared to control. However, the time spent in the OA was not significant in the F2 generation (p = 0.216, 95 % CI −0.92, 3.54). The difference between mean OA entries (%) and time spent in OA (%) in heroin and control group in F3 generation, on the other hand, was not statistically significant.

Effects of paternal heroin exposure which were investigated using elevated plus maze (EPM) test indicated no significant differences for the open arms (CA) entries between the heroin exposed rats with its offspring (One-way ANOVA, *p* > 0.001). However, a significant difference was seen between the heroin exposed rats and its third generation (F3). This shows that the behavior pattern was passed down only to F1 and F2 but not to its subsequent generation.

### Effects of paternal exposure of heroin on the percentage of time spent and entries into the central zone of arena in the open field (OF) test

**Fig. 5:**
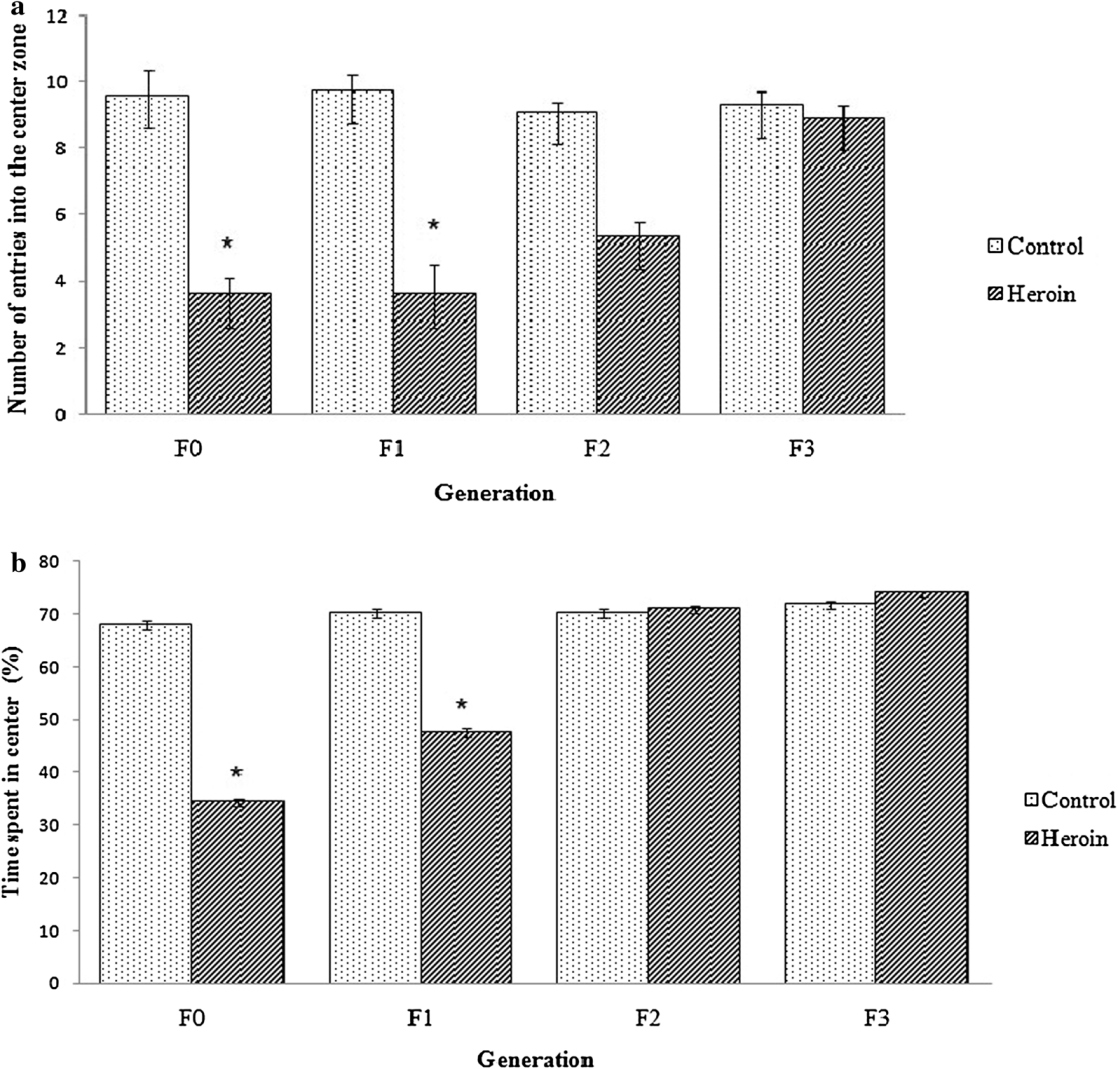
Number of entries in the center (**a**) and percentage of time spent in the center (**b**) in the open field test. Values are mean ± SEM. *Statistically significant difference compared to the control group (p < 0.05)

Number of entries into the central zone and percentage of time spent in the central zone are presented in Fig. 5. Heroin exposed rats showed statistically significant decrease (p = 0.001, 95 % CI 4.15, 7.85) in the number of entries into the central area compared to the control group (Fig. 5a). In addition, the offspring of F0-heroin rats exhibited significantly lower entries into the central area (p = 0.01, 95 % CI 1.27, 7.72), as compared to F1-control. Furthermore, a statistically significant difference was also observed between the second generations of the heroin exposed rats and control rats (F2-heroin and F2-control) in the number of entries in the center of the OF (p = 0.017, 95 % CI 0.26, 2.23). This pattern however was not seen in F3 generation, which showed no statistically significant difference between F3-heroin and F3-control in both parameters measured.

Percentage of time spent in the center of OF is depicted in Fig. 5b. F0-heroin rats spent less time in the center zone as compared to F0-C during withdrawal phase (p = 0.001, 95 % CI 31.51, 35.31). Interestingly, F1-heroin also exhibited significantly shorter time spent in the center of OF than the F1-control (p = 0.001, 95 % CI 31.04, 34.29). In contrast, no statistical differences were found on the time spent by rats in the central square between F2-heroin and F2-control (p = 0.39, 95 % CI −2.29, 0.96). Both groups of the F3 generation exhibited no significant differences, indicating that the anxiety pattern has normalized towards control.

It was found that anxiety behavior between the F0-heroin and F1-heroin groups were similar (post hoc*, p* > 0.005), implicating that transgenerational anxiety effects from the paternal to the descendants sustained over two generations.

### Effects of paternal exposure of heroin in resident intruder (RI) test

Long-term behavioral effects of exposure to heroin in the first generation (F0) were then evaluated. Table 2 shows behavior phenotypes that were observed in this study: non-social activities, social activities, and aggressive behavior. Heroin exposed rats exhibited a pattern of pathological aggression as shown in tail swish, lateral threat, bite, and clinch/bite behavior during adulthood in RI test (p < 0.005). In non-social parameters, significant differences were exhibited in attention, rear, and body care behavior with heroin exposed male rats displaying higher frequency of these behaviors (p < 0.05). In social parameters, significant differences between the heroin exposed rats and control rats were displayed in approach, walk away, and social sniff (p < 0.05). In addition, in the aggression parameter, F0-heroin and F0-control exhibited a significantly difference in all of the parameters observed (p < 0.05), with F0-heroin displayed a higher frequency displaying the behaviors.

**Table 2:** Behavioral observations from resident intruder test showing the mean for each group

Behavior	Frequency							
	F0-C	F0-H	F1-C	F1-H	F2-C	F2-H	F3-C	F3-H
Non-social activity								
Attention	30.62	24.83*	30.06	24.17*	30.50	24.17*	29.31	27.41
Rear	0.62	5.75*	0.87	5.5*	1.57	5.71*	0.88	1.31
Sniff	23.0	20.87	22.87	21	23.13	19.87	20.11	20.72
Walk	22.50	22.25	22.87	21.62	22.37	21.87	23.3	22.92
Body care	1.25	4.87*	1.37	5.1*	1.50	5.12*	2.31	6.22*
Social activities								
Approach	8.50	20.37*	7.5	21.62*	8.25	21.0*	9.22	15.51*
Follow	1.87	1.62	2.37	1.28*	4.0	2.25*	2.35	3.42
Walk away	2.50	10.12*	2.50	9.87*	2.62	0.62	3.16	4.26
Social sniff	23.25	39.62*	23.0	40.50*	23.0	28.12	23.16	27.17*
Genital sniff	8.62	9.87	8.75	10.50	7.37	10.62*	8.65	8.88
Mount	0.63	1.37	0.63	1.50	0.75	0.87	0.71	0.91
Aggression								
Tail swish	0.75	3.75*	1.25	4.12*	1.25	3.87*	0.65	1.31
Lateral threat	0.37	3.62*	0.63	3.63*	0.37	3.62*	0.39	0.61
Bite	2.37	9.25*	2.37	9.50*	2.87	9.12*	2.73	5.28*
Clinch/fight	1.25	7.5*	1.50	7.50*	1.62	2.50	1.45	3.32*

Data are given as mean frequencies. Student’s *t* test: the asterisk means statistically significant difference compared to control rats (*p* < 0.05)

It was then further evaluated whether the next generation would also be affected by the previously heroin exposed father. Rats in the second generation (F1-heroin) showed higher frequency in non-social and social activity than the controls. In addition, the F1-Heroin also exhibited an increase in aggression parameters observed. This behavioral pattern was also observed in the third generation, F2-Heroin. However, in F3 generation, lower aggression behavior was observed.

As shown in Table 3, F0-heroin rats not only attack its intruder frequently, but also showing longer duration of attack towards the intruder and made a significantly longer time in clinch and fight. This pattern was exhibited by F1-heroin and F2-heroin. However, F3-heroin displayed a shorter time of attack towards the intruder, almost similar to F3-control.

**Table 3:** Duration of the ethological measures

Behavior	Duration (s)							
	F0-C	F0-H	F1-C	F1-H	F2-C	F2-H	F3-C	F3-H
Non-social activity								
Attention	86.75	72.00	85.50	68.75*	86.75	68.75	71.83	70.05
Rear	1.63	7.13*	1.50	7.13*	1.31	7.00	5.79	6.22
Sniff	70.00	42.13*	69.00	41.88*	68.25	39.88	45.25	42.41
Walk	39.50	32.13	39.13	31.35	39.13	34.63	34.86	34.54
Body care	2.46	19.13*	1.51	19.20*	2.39	19.63*	16.05	17.57
Social activities								
App	9.00	20.13*	10.06	20.15*	9.50	20.00*	18.13	19.08
Follow	1.47	1.38	1.66	1.53	2.06	1.75	1.97	2.07
Walk away	1.94	12.56*	2.30	12.71*	2.51	2.47	10.70	11.64
Social sniff	88.63	161.38*	94.63	162.13*	86.88	78.88	144.94	150.52*
Genital sniff	29.13	48.50*	30.50	49.46*	29.75	52.00*	47.60	49.80
Mount	1.49	3.50	1.49	3.40	1.49	1.51	1.75	1.63
Aggression								
Tail swish	0.61	5.23*	0.94	5.43*	1.00	5.29*	4.60	5.03
Lateral threat	0.56	3.13*	0.93	3.08	0.31	3.17*	2.53	2.73
Bite	2.81	8.74*	2.45	8.25*	2.59	7.41	6.41	6.71
Clinch/fight	0.88	23.00*	1.21	23.00*	0.96	23.75*	19.14	21.14

Durations taken for the behavioral observations from resident intruder test showing the mean for each group. Student’s *t* test: the asterisk means statistically significant different from control rats (*p* < 0.05)

## Discussion

Drug abuse is an increasing societal burden. While it has been well documented that maternal opioids abuse during pregnancy results in detrimental effects in the offspring, less is understood about the contribution of paternal drug abuse on the next generation. The present study focused on paternal effects of heroin exposure during adolescence towards progenies i.e. F1, F2, and F3 generations.

The results of the present study demonstrated that heroin exposure produced adverse effect on the litter sizes (F1) as compared to saline administered group. The litter sizes of heroin treated rats were significantly lower than in control group (mean pup = 10). Our results were similar to other studies on fetal effects of opiate and other toxicants administration that caused abnormalities in their offspring such as decreased in the size of litters and lost in weight [14, 16–18]. Upon entering the human body, heroin is metabolized into 6-acetylmorphine (6-a. m.) and morphine, which will then be converted to morphine-3-glucuronide and morphine-6-glucuronide [19]. One of the possibilities that may leads to this paternal effects is that morphine may act directly as a mutagen on sperms, but the mechanisms of action has yet to be determined. In addition, the viability of sperms might also be affected after exposure to heroin [20]. Accumulation of the mu opioid receptors as well as endogenous expression of beta-endorphin in the male reproductive tract proposed that paternal gametes are receptive to endogenous and exogenous opioids [21]. μ-, κ-, and ∆ opioid receptors are present in oocytes, most likely for mediating oocyte maturation [22]. Hence, the presence of opioid receptors on gametes not only maintains proper functions and development, but may lead to transgenerational inheritance [23]. Several previous studies have demonstrated that drug abusing behavior could lead to subtle mutations in the sperms [16, 24–26]. Furthermore, it has been determined previously that drugs and other toxicants will accumulate in the semen and some may even bind to receptors on the sperms [27–30]. These studies suggested that abused substances may cause alterations in sperms thus influencing the development of the offspring, or transported to the ova via the seminal fluid, or by directly binding to the sperms and then affecting the development of the conceptus.

Research has shown that prenatal exposure to drugs of abuse can have long-term effects on the behavior of offspring [31]. Most of the previous studies focused on the impact of exposure of mothers to opioids such as heroin, morphine, and codeine during pregnancy, and its possible effects on prenatal opioid exposed offspring [32–34]. Other studies on congenital deficiencies, behavioral disorders, learning and memory impairment in offspring of addicted fathers have also been reported [11, 35]. This current study provides additional information that highlights the impact of heroin exposure of the father up to four generations of the offspring in terms of anxiety and aggressive behavior. OF and EPM are commonly used to measure anxiety-related behaviors in laboratory rodents. In EPM, anxiety-like behaviors are characterized by a lower frequency of entries and also shorter time spent in the open arms. To further validate the anxiety behavior, rats were then tested in the OF test. In OF, the anxiety behavior is characterized by a lower percentage of entries and shorter time spent in the center zone of the arena. Our results indicated that heroin addicted male rats were more anxious in both the OF box and EPM tasks which are in accordance with other studies using female addicted rats [11, 33]. Repeated heroin use leads to changes in the anatomy and physiology of the brain, consequently affecting the abilities in decision making, self-regulation behavior and response to stressful situations (Drug Policy Alliance, 2016). Additionally, it could lead to significant levels of tolerance and physical dependence. In this study, anxiety behavior in heroin induced male rats and its male progenies were increased as compared to the control rats. The offspring of the heroin exposed fathers displayed differences in several measures of behavioral tasks that look into anxiety level during the EPM and OF tests. Higher entries in the closed arms that were observed in the present study were also reported by Ahmadalipour et al. [36] using morphine induced female rats. We have further evaluated the anxiety level of the second generation of male heroin addicted rats. F2-heroin exhibited a statistically significant lower percentage of entries in the open arm of the EPM compared to F2-control. Interestingly, there was no significant difference in time spent in the OA between the two groups. The same pattern was also seen in the OF test. This indicates the rats of the F2-Heroin have an increase in exploratory behavior in the novel environment that has been introduced, despite the lower entries in the open arms and open arena. Notably, the anxiety levels in the third generation were reduced as compared to their father.

Anxiety is generally believed to be associated with aggressive behavior [37]. Thus, we evaluated whether there is an association between aggression and anxiety-like behavior in an animal model. The current findings demonstrate significant transgenerational effects of male rats addicted with heroin in the RI test. Heroin addicted rats are more aggressive than the controls in the RI test with a significant difference on several parameters that measures aggression i.e. tail swish, lateral threat, bite and clinch. These effects occurred in the absence of any direct exposure to heroin, and provide evidence that its administration may give psychological impacts on the addicts. From a pharmacological perspective, aggressive behavior can be escalated either by low acute heroin doses or during withdrawal from prolonged administration to repeated high heroin doses, presumably based on separate neural mechanisms. Interestingly, the male offspring of the heroin exposed rats displayed more aggressive responses towards the intruders when compared with the age-matched offspring of saline administered rats. This can be suggested as transgenerational effects of adolescent drug use, even in the absence of continued use. This pattern can also be observed in the F2 generation but certain behaviors were not significantly different with the F2-control. However, the F3 generation of the heroin administered rats displayed a much lower aggressive response as compared to the previous generations.

Interestingly, rats that displayed heightened anxiety-like behavior exhibited higher aggression level, as indicated by the reduced time spent in the open arms of the EPM test as well as decreased ambulatory activities in the OF test. The mechanisms of heroin exposure towards the evolutionary phase of the gestational period and offspring’s anxiety and aggression level remains unknown and needs further investigation. Many drugs including morphine may be mutagenic which often cause a minor but significant change in the physiological parameters, behaviors and neuroendocrine parameters [38].

In previous studies, possible effects of addicted father towards children were less considered. Thus, the results of this current study can be one of the supporting data highlighting the effects of heroin addicted fathers on children. It can be postulated that epigenetic mechanisms could be involved in transgenerational effects of heroin exposed paternal rats.

Epigenetic factors such as histone modifications (e.g. acetylation) have been implicated in the behavior of addiction [39]. In some instances, histone modifications alter the accessibility of DNA, hence affecting gene expression. DNA methylation (the addition of a methyl group to cytosine nucleotides, converting them to 5-methylcytosine) is another epigenetic process that has been shown to be involved in modulating behavior in response to substance abuse. For example, DNA methylation as well as histone acetylation represent key factors in regulating the *BDNF* gene, which encodes brain-derived neurotrophic factor (BDNF), a protein that is involved in numerous neurological conditions such as schizophrenia, depression, epilepsy, Alzheimer’s disease, obesity and drug addiction at many stages of development [40].

As yet, no study has tracked the epigenetic effects on behavior of descendants of heroin abused fathers through the generations. Research is underway to examine the epigenetic influences on longstanding aggression behavior as a result of transgenerational substance abuse.

## Conclusions

The idea that maternal heroin abuse can impact phenotype of subsequent generations is not new. Data from our studies however, highlighted the effects of paternal heroin exposure on progenies. Our findings have shown that heroin exposure can affect the levels of anxiety and aggression in the F0 generation. Of note, these effects were also observed in both the first (F1) and second (F2) generations, suggesting multigenerational and transgenerational effects triggered by adolescent exposure to heroin. Further investigations by our group are currently under way to understand the mechanisms involved in these observed effect.

## Abbreviations

°C: degree celcius
a.m.: ante meridiem
ANOVA: analysis of variance
EPM: elevated plus maze
F0: first generation
F1: second generation (offspring of F0)
F2: third generation (offspring of F1)
F3: fourth generation (offspring of F2)
g: gram
h: hour
kg: kilogram
ml: mililiter
mg: miligram
OF: open field
PND: postnatal day
RI: resident intruder
S.E.M: standard error mean
